# Evaluating the Potential of Four Native *Ziziphus* Species for Restoring Savanna‐Like Habitats in Jinsha River Under Climate Change

**DOI:** 10.1002/ece3.74531

**Published:** 2026-10-28

**Authors:** Yulin Xu, Zaiqiu Xiong, Zhiming Zhang, Gao Chen

**Affiliations:** ^1^ Key Laboratory of Phytochemistry and Natural Medicines Chinese Academy of Sciences Kunming China; ^2^ Yunnan Key Laboratory for Integrative Conservation of Plant Species With Extremely Small Populations Kunming Institute of Botany, Chinese Academy of Sciences Kunming China; ^3^ School of Ecology and Environmental Sciences Yunnan University Kunming China

**Keywords:** climate change, ecological restoration, heteromorphic seeds, Jinsha River, MaxEnt, *ziziphus*

## Abstract

The Jinsha River, as the upper section of the Yangtze River, plays a key role as an ecological barrier, yet its dry‐hot valleys face severe degradation due to harsh climate and human activities. Four native *Ziziphus* species ( *Z. mauritiana* , *Z. montana* , *Z. mairei*, and *Z. xiangchengensis*) were selected to assess their potential for restoring “valley‐type savanna” vegetation through field investigations, species distribution modeling, germination experiments, and interviews. Field investigations showed that *Z. mairei* and *Z. xiangchengensis* exhibit limited distributions and small populations, requiring artificial propagation and protection. MaxEnt models performed well (AUC > 0.9). *Ziziphus montana* occurs in northwestern Yunnan and is primarily limited by temperature seasonality (bio4), elevation, and coldest quarter temperature (bio11). Under future climate scenarios, its suitable habitat is projected to contract by 11.3%–20.4%, with the remaining highly suitable area retreating toward the Jinsha River basin. Bio11 is the primary factor influencing the distribution of *Z. mauritiana* , and its suitable range is projected to expand by 44.1%–119.1%. Seed heteromorphism was documented for the first time in *Z. xiangchengensis*, *Z. montana* , and *Z. mairei*, classified into Type A (2 carpels) and B (3 carpels) based on carpel number, with Type A seeds dominating (66.0%–94.8%). These two seed types showed significant differences in temperature response and dormancy characteristics. Four *Ziziphus* species displayed strong physical dormancy, removing the endocarp and cracking the seed coat results in a 100% germination rate, providing a practical solution for large‐scale seedling propagation. Based on assessment, *Z. montana* is more suitable for ecological restoration in the Jinsha River Basin. The population age structure is of the expanding type and serves medicinal, edible, agricultural, ecological restoration, and handicraft purposes. We advocate for community‐based “interactive” ecological restoration, using economic incentives to drive ecological recovery. This study contributes to ecological restoration, germplasm conservation, and local economic development.

## Graphical abstract

This study predicted the current and future distributions of four native *Ziziphus* species in the Jinsha River Basin, and also investigated their seed dormancy and germination characteristics. Based on a comprehensive assessment, *Z. montana* is the most suitable species for ecological restoration in the Jinsha River Basin, exhibiting high and stable habitat suitability as well as multiple uses. We report for the first time seed heteromorphism in *Z. montana* , *Z. mairei*, and *Z. xiangchengensis*, which differ in germination and dormancy; we hypothesize that this phenomenon represents an adaptation to the highly heterogeneous environment of the dry river valley and may also have a phylogenetic basis.

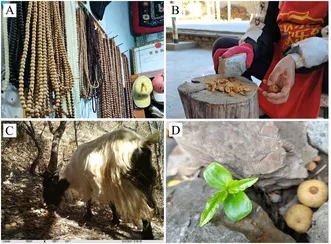

## 1 Introduction

The Jinsha River, as the upper section of the Yangtze River in Southwest China, serves as a critical ecological barrier (Li et al. 2020). The dry‐hot valleys of this region are characterized by extreme aridity, high temperatures, low precipitation, and high evaporation, leading to a severe imbalance between heat and water availability (Sun et al. 2025). The predominant vegetation type is the “valley‐type savanna vegetation,” which establishes this area as a vital repository of non‐zonal vegetation types and a floristic gene pool in China (Zhu et al. 2020). However, due to the interplay of complex topography, harsh climate, and human interference disturbances (cultivation, overgrazing, deforestation, and hydropower construction), this region has become one of the most severely affected by soil erosion and faces the greatest challenges in vegetation restoration in the country. This area is thus a typical vulnerable coupled natural‐social system (Li et al. 2020; Zhang et al. 2025). Consequently, systematic ecological restoration within the Jinsha River basin is urgently required. The fundamental principle for vegetation restoration in such fragile ecoregions is to prioritize natural regeneration, supplemented by artificial restoration, with strategies tailored to local conditions based on scientific assessment (Gann et al. 2019). Trees constitute the iconic framework of the savanna landscape, are pivotal in creating localized fertile islands and enhancing biodiversity, and serve as living gene pools reflecting plant adaptation to extreme dry‐hot environments (Zhang et al. 2025). Therefore, employing native tree species that are tolerant of poor soils, drought, and heat is fundamental to the success of ecological restoration.

The genus *Ziziphus* comprises approximately 100 species worldwide, with 18 occurring in China. It has a pantropical distribution, primarily in tropical, subtropical, and warm‐temperate regions of Asia and the Americas (Paudel et al. 2023). It is thought to have originated in Asia, with subsequent migration to Africa giving rise to open grasslands and deserts (Rickenback et al. 2024). The genus *Ziziphus* is economically important (Li et al. 2010) and serves as a quintessential multipurpose plant for food, medicine, ecological protection, and religious practices. Most *Ziziphus* fruits are edible and rich in vitamins, amino acids, polysaccharides, minerals, polyphenols, and flavonoids and can be eaten fresh or processed into various products (Chen and Tsim 2020; Stan et al. 2022). The seeds contain saponins, flavonoids, and alkaloids with antibacterial, anti‐inflammatory, and antioxidant activities (Shahrajabian et al. 2019), and have a long medicinal history in many Asian countries (Chen and Tsim 2020; Paudel et al. 2023). The leaves are a nutritious forage for livestock and are also used in traditional medicine (Gupta 1993; Damiano et al. 2017). The stony endocarp is used to make prayer beads in China, Nepal, and Bhutan (Dorji et al. 2015; Pathak et al. 2017). The dense, hard wood (535–1080 kg/m^3^) is used for agricultural implements, charcoal, and furniture (Paudel et al. 2023). Shrubby, thorny species can also be planted as hedges, and the plants serve as nectar sources for honey production (Kheloufi et al. 2020; Grice 1996). In summary, *Ziziphus* species are important multipurpose trees of significant economic value.

Beyond their economic value, *Ziziphus* species also possess significant ecological value. With well‐developed root systems, strong root‐suckering ability, and tolerance to drought, nutrient‐poor soils, high temperatures, and salinity, they are highly adaptable pioneer species for ecological restoration, providing wind protection, soil conservation, and wildlife habitat (Shahrajabian et al. 2019; Pandey et al. 2010). *Ziziphus spina‐christi* tolerates winter temperatures as low as −10°C and summer temperatures as high as 50°C (Bahwireth et al. 2023). Of the 18 *Ziziphus* species in China, *Z. mauritiana* is widespread across southern China and common in the Jinsha River basin, whereas *Z. xiangchengensis*, *Z. montana* , and *Z. mairei* are endemic to this region with restricted distributions. *Ziziphus mairei* is IUCN Endangered, found at 1900–2000 m in scrub and riparian forests from central to northwestern Yunnan (Wang et al. 2023). *Ziziphus xiangchengensis* is extremely limited, mainly to Xiangcheng (Sichuan, China) and central Nepal (Pathak et al. 2017). *Ziziphus montana* occurs in northwestern Yunnan, Sichuan, and Tibet. The three endemics are closely related (Li et al. 2010), but research on them is scarce and their ecological potential unexplored. In contrast to *Z. mauritiana* , for which seed germination, nutrition, and pests have been relatively well studied (Grice 1996; Bebawi et al. 2016). *Ziziphus* seeds via fruits dispersed by cattle, goats, donkeys, horses, *Cebus apella* , *Ateles paniscus* , and foxes (Zhang and Wang 1995; Grice 1996; Bebawi et al. 2016). Its seeds commonly exhibit both physical and physiological dormancy (Finch‐Savage and Leubner‐Metzger 2006). To better understand their survival and regeneration strategies, we examined seed germination and dormancy in the four native *Ziziphus* species and assessed their potential for ecological restoration in the Jinsha River basin.

Seed heteromorphism is the production of two or more distinct seed types by a single plant, differing in morphology, physiology, or ecological function (Venable 1985). Our preliminary investigation indicate that *Z. montana* , *Z. xiangchengensis*, and *Z. mairei* exhibit this trait. Heteromorphic seeds are predominantly found in disturbed habitats such as arid and semi‐arid regions, deserts, and saline soils, and are most common in annual plants (Ellner and Shmida 1981; Baskin et al. 2014). This phenomenon serves as a “bet‐hedging” strategy that spreads offspring survival risks across space and time, enhancing long‐term fitness in variable environments (Venable 1985; Imbert 2002; Scholl et al. 2020). Seed heteromorphism has been documented in 292 angiosperm species across 129 genera and 26 families, most frequently in Asteraceae, Chenopodiaceae, and Fabaceae (Wang et al. 2010). However, reports from woody plants remain rare. This study presents the first report of heteromorphic seeds in *Z. montana* , *Z. xiangchengensis*, and *Z. mairei*, suggesting that this trait may represent an adaptive strategy to the highly heterogeneous environments of the Jinsha River basin.

*Ziziphus* species are multipurpose plants with considerable potential for development in arid regions and barren areas such as the Jinsha River Basin. To comprehensively evaluate the restoration potential of four native *Ziziphus* species, we aim to progress from habitat modeling and propagation biology to community‐driven restoration. Specifically: (1) using MaxEnt modeling to predict current and future suitable habitats, thereby identifying species adapted to the arid Jinsha River basin and priority areas for restoration; (2) quantifying heteromorphic seed types and their proportions; (3) assessing temperature responses and dormancy‐breaking requirements of heteromorphic seeds to enable rapid seedling production for restoration; (4) investigating the population age structure composition, seed dispersers, and ethnobotany of *Z. montana* in order to explore economic incentives that can engage local communities in restoration efforts.

## 2 Methods

### 2.1 Research Object and Region

Four *Ziziphus* species distributed in the Jinsha River Basin were selected: *Z. mauritiana* , *Z. montana* , *Z. mairei*, and *Z. xiangchengensis*. As native trees of the local savanna vegetation, they are considered key species for evaluating restoration potential in this region. Mature fruits were collected and air‐dried in a ventilated area for subsequent experiments. Sampling details and regional climate data are provided in Table S1.

### 2.2 Constructing Species Distribution Models

The occurrence data for the four *Ziziphus* species was compiled from three sources: field investigations, herbarium specimens, and photographic records. Initially, duplicate and incomplete data were manually eliminated. Subsequently, 1‐km buffers were generated around occurrence points in ArcMap 10.8 (Esri, Redlands, CA, USA), retaining only one record per grid cell to reduce sampling bias, spatial autocorrelation and overfitting in MaxEnt (version 3.4.4) model (Boria et al. 2014). A total of 221 distribution points for *Z. mauritiana* and 38 for *Z. montana* were retained for modeling. The *Z. xiangchengensis* and *Z. mairei* were not analyzed, as the small number of distribution points (only 11 and 8, respectively) was insufficient for constructing accurate models.

Current and future (2041–2060) climate and elevation data were sourced from the World‐Clim database, with a selected resolution of 30 arc‐seconds (https://www.worldclim.org/). Two Shared Socioeconomic Pathways, namely SSP126 and SSP585, from the BCC‐CSM2‐MR climate model developed by the Beijing Climate Center, China Meteorological Administration, were selected. These two pathways represent two contrasting future development scenarios, with one of low‐carbon sustainability and another of high fossil‐fuel dependency (Gillett et al. 2016). The global climate data were then clipped to the extent of China and exported to ASCII format using ArcMap. Environmental variables and species occurrence data were imported into the MaxEnt to screen for and identify the most important bioclimatic factors. The data was partitioned into 75% for training and 25% for testing. The model was run with 5000 iterations and replicated 20 times (Phillips et al. 2006). Environmental variables with a contribution of less than 1 were iteratively removed based on the preliminary modeling results. This process was repeated until the contribution of all modeling factors in the model is greater than 1%. Subsequently, climatic data were extracted for the species occurrence points, and pearson correlation analysis was conducted on the selected environmental variables using SPSS (Version 27.0, IBM Corp., Armonk, NY, USA). For any environmental factor with a correlation greater than 0.8, only the one with the higher contribution is retained (Shen et al. 2021). Furthermore, the retained environmental variables were selected based on their ecological relevance to the physiology and geographic distribution of the *Ziziphus* species. Final models were constructed using the refined set of environmental variables. The percent contributions of various environmental variables to the MaxEnt models for *Z. mauritiana* and *Z. montana* are presented in Table 1. The robustness of the MaxEnt model was evaluated using the Area Under the Curve (AUC) of the Receiver Operating Characteristic (ROC) curve (Phillips et al. 2006). An AUC value greater than 0.9 indicates high predictive accuracy and reliability of the model (Swets 2013). The MaxEnt results were imported into ArcGIS and reclassified into five habitat suitability categories: highly suitable (0.6–0.8 and 0.8–1.0), moderately suitable (0.4–0.6), lowly suitable (0.2–0.4), and unsuitable (0–0.2). Finally, the area of each suitability category was calculated.

**TABLE 1:** The contribution rate of the MaxEnt model of environmental variable modeling.

Environmental variable	Description	Percent contribution (%)	Permutation importance (%)	Units
*Z. mauritiana*				
bio11	Mean temperature of coldest quarter	81.0	52.4	°C × 10
bio4	Temperature Seasonality	6.3	2.3	standard deviation ×100
bio3	Isothermality	5.1	6.9	×100
bio5	Maximum temperature of warmest month	2.1	1.4	°C
bio2	Mean Diurnal Range	1.7	7.3	°C
bio8	Mean temperature of wettest quarter	1.4	2.9	°C
bio7	Temperature annual range	1.2	1.8	°C
elev	Elevation	1.1	25.0	m
*Z. montana*				
bio4	Temperature Seasonality	27.3	55.4	×100
elev	Elevation	21.6	8.5	m
bio11	Mean temperature of coldest quarter	17.4	25.1	°C
bio12	Annual precipitation	14.8	0.3	mm
bio3	Isothermality	14.2	0.1	×100
bio14	Precipitation of driest month	2.3	4.8	mm
bio15	Precipitation seasonality	1.4	1.9	mm
bio2	Mean Diurnal Range	1.0	3.9	°C

### 2.3 Heteromorphic Types of Four *Ziziphus* Species

For each species, 250 mature fruits were selected and randomly divided into five groups. After removal of the exocarp and pulp, fruits were soaked for 3 days at room temperature. Cleaned endocarps were dissected longitudinally and transversely to record seed types and counts. Based on these observations, the heteromorphism in *Ziziphus* was identified as seed dimorphism, consisting of Type A (2 carpels) and Type B (3 carpels).

### 2.4 Effect of Temperature on the Germination of Four *Ziziphus* Species

To evaluate seedling production capacity for restoration, we tested germination under field‐simulated temperatures (constant 20°C and alternating 15/25°C). Seeds of the four *Ziziphus* species were extracted and separated by heteromorphic type (A/B). For each type, 120 seeds were randomly divided into six groups of 20 seeds. After rinsing four times with sterile water, each group was placed on 1% agar plates and incubated in growth chambers. All groups were subjected to a 12‐h light/12‐h dark photoperiod, with three replicates for each temperature regime. Germination was recorded daily, and the experiment was terminated when no germination occurred for ten consecutive days. Germination was defined as radicle protrusion to half the seed length. A two‐way ANOVA was performed using SPSS to evaluate the main effects of temperature and seed type and their interaction, followed by Fisher's LSD test for multiple comparisons.

### 2.5 Breaking Dormancy of Four *Ziziphus* Species

To test the effect of pulp on germination, both pulped and intact fruits of the four species were sown in nursery pots (three replicates of 20 fruits each) and watered weekly for 6 months. No germination occurred in either treatment, indicating physical dormancy.

Therefore, we conducted the experiments directly with seeds. The experiments were conducted to investigate seed dormancy types and to identify practical methods for breaking dormancy, with the aim of facilitating large‐scale seedling production. Four treatments were applied based on optimized methods and practical considerations from other *Ziziphus* species (Hassen et al. 2005; Maraghni et al. 2010; Bebawi et al. 2016): seed coat scarification, 1% GA_3_ (6 h), hot water (70°C, cooled to room temperature), dry storage (1‐year). To test whether goat digestive passage promotes germination (following preliminary observations that goats consume *Z. montana* fruits), we artificially fed goats with the fruits and collected regurgitated pits. Seed handling and observations followed Section 2.4. Germination indices were calculated using the ‘germinationmetrics’ package, and treatment differences were analyzed using one‐way ANOVA with Fisher's LSD test for multiple comparisons.

### 2.6 Population Renewal Strategy of *Ziziphus montana*

*Ziziphus montana* was selected for detailed investigation because it is projected to maintain high habitat suitability under both current and future climates, and its population size is moderate compared to the rare *Z. xiangchengensis* and *Z. mairei*. This makes it a suitable representative for assessing population regeneration status, value, seed dispersal, and the potential for community‐based restoration.

Field surveys and community interviews were conducted in populations of *Z. montana* at Jiangbian (27°16′55″ N, 100°13′54″ E) and Peiliang (27°17′16.85″ N, 100°14′18.25″ E) villages, selected using herbarium records and species distribution modeling. A 20 × 20 m quadrat was established at each site, and all individuals were enumerated. For each plant > 1.3 m tall, we recorded basal diameter (at 30 cm above ground), crown width, height, and threat status. Semi‐structured interviews (*n* = 55) were conducted with local residents to record historical uses and perceived population trends of *Z. montana* (Table S2). A total of 25 quadrats were surveyed. Age classes were defined by basal diameter (BD) at 5‐cm intervals (Class I: < 5 cm; Class XI: ≥ 50 cm). Statistical analysis and graphical representation were then conducted.

Seed dispersers were monitored using three infrared cameras per site, deployed in areas of high fruit fall density throughout the fruiting season (October 2020—January 2021). Cameras operated continuously (24 h/day) at low sensitivity; each trigger event captured three photographs and a 30‐s video, with consecutive triggers permitted.

## 3 Results

### 3.1 Distribution and Ecological Adaptability of Four *Ziziphus* Species

#### 3.1.1 Analysis of Environmental Variables

*Ziziphus xiangchengensis* and *Z. mairei* were excluded from MaxEnt modeling due to insufficient occurrence records (11 and 8, respectively) for reliable model construction. The AUC values for the species distribution models of both *Z. mauritiana* and *Z. montana* exceeded 0.9 (Table S3), indicating that the model performance was reliable and suitable for subsequent analysis (Swets 2013). As shown in Table 1, bio11, bio4, and bio3 were identified as the key environmental factors influencing the distribution of *Z. mauritiana* , with cumulative contributions of 92.4% in percent contribution and 61.6% in permutation importance. The key environmental factors influencing the distribution of *Z. montana* were identified as bio4, elev, and bio11, with cumulative percent contribution and permutation importance values of 66.3% and 89%, respectively. According to the univariate analysis (Figure S1), bio11, bio4, and bio7 carried significant weight in modeling *Z. mauritiana* , whereas bio4, bio3, and elev were the dominant factors for *Z. montana* . The bio11 and bio4 were identified as the most suitable single predictors for *Z. mauritiana* and *Z. montana* , respectively, with both variables containing substantial unique information not present in other environmental factors.

#### 3.1.2 Current and Future Distribution

The current distribution of *Z. xiangchengensis* is highly restricted, with confirmed occurrences only in Dongsong Township and along the banks of the Shuoqu River in Xiangcheng County, Ganzi Tibetan Autonomous Prefecture, Sichuan Province. The documented distribution of *Z. mairei* is also limited, with confirmed occurrences only in Kunming, Kaiyuan, and Wenshan. According to the China Biodiversity Red List, *Z. xiangchengensis* is classified as Near Threatened (NT), while *Z. mairei* is assessed as Endangered (EN) and requires corresponding protection. The current and future potential distribution areas of *Z. mauritiana* and *Z. montana* are shown in Figure 1, with the corresponding areas of suitable habitats listed in Table 2.

**FIGURE 1:**
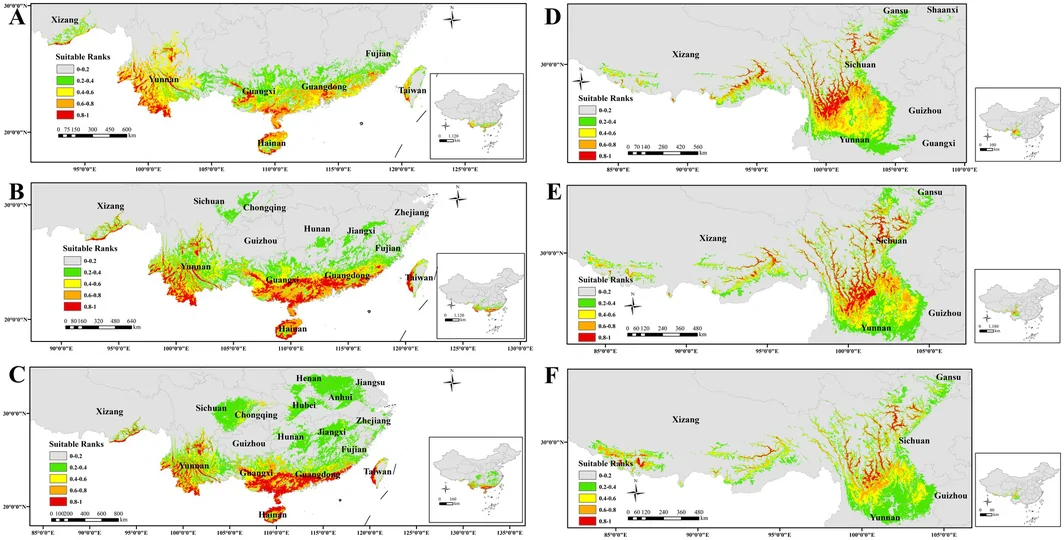
Predicted current and future (2050s) distribution maps for *Z. mauritiana* (A–C) and *Z. montana* (D–F). (A, D) Current distribution; (B, E) projections under the SSP1‐2.6 scenario; (C, F) projections under the SSP5‐8.5 scenario.

**TABLE 2:** Suitable habitat for *Z. mauritiana* and *Z. montana* .

	Scenario	Area (×10^4^ km^2^)				
		Suitability of habitat				
		Low (0.2–0.4)	Moderate (0.4–0.6)	High (0.6–0.8)	High (0.8–1.0)	Total
*Z. mauritiana*	Current	17.22	14.87	12.45	4.19	48.73
	SSP1‐2.6	28.44	14.86	13.97	12.93	70.21
	SSP5‐8.5	60.79	16.18	12.97	16.85	106.79
*Z. montana*	Current	14.33	10.96	6.12	4.02	35.44
	SSP1‐2.6	14.62	8.24	5.02	3.56	31.44
	SSP5‐8.5	15.95	7.15	3.54	1.58	28.22

For *Z. mauritiana* , the current highly suitable habitat covers approximately 16.64 × 10^4^ km^2^ and is concentrated in warm areas such as Yunnan, Hainan, southern Guangxi, southern Guangdong, western Taiwan, and the Zhangzhou‐Xiamen area of Fujian (Figure 1A). The moderately suitable habitat (14.87 × 10^4^ km^2^) is spatially adjacent to the highly suitable regions, while the lowly suitable habitat extends into cooler areas within these provinces. Under future climate scenarios, the highly and lowly suitable habitats for *Z. mauritiana* are projected to expand significantly, with the magnitude of increase positively correlated with emission levels. Under the SSP1‐2.6 and SSP5‐8.5 scenarios for the 2050s, the highly suitable habitat is projected to reach 26.9 × 10^4^ km^2^ and 29.82 × 10^4^ km^2^, representing an increase of 61.66% and 79.21%, respectively (Table 2). Notably, most of the current moderately suitable habitat is projected to transition into highly suitable as temperatures rise, reflecting the species' preference for warmer climatic conditions.

For *Z. montana* , the current highly suitable habitat covers approximately 10.14 × 10^4^ km^2^. The highly suitable habitat exhibits a distinct distribution pattern, concentrated in northwestern Yunnan (Lijiang and Diqing city) within the Jinsha and Lancang River watersheds, and extending to northern Panzhihua and eastern/western Liangshan (Sichuan) within the Yalong River watershed, a tributary of the Jinsha River (Figure 1D). The moderately suitable habitat covers approximately 10.96 × 10^4^ km^2^, distributed across northern Dali, northern Chuxiong, northern Kunming, western/southern Zhaotong, and southern Liangshan. Under future climate scenarios, both the highly and moderately suitable habitats for *Z. montana* are projected to decrease, while the lowly suitable habitat may expand slightly. The highly suitable habitat of *Z. montana* declines to 8.58 × 10^4^ km^2^ under SSP1‐2.6 and 5.12 × 10^4^ km^2^ under SSP5‐8.5, corresponding to negative changes of 15.38% and 49.51% (Table 2). Under higher emission scenarios, this contraction becomes particularly pronounced. Nevertheless, the remaining highly suitable habitat remains predominantly concentrated within the Jinsha River and its tributary watersheds, indicating that this region will continue to serve as a climatic refuge for the species.

### 3.2 Heteromorphic Types of Four *Ziziphus* Species

Seed heteromorphism was observed in *Z. xiangchengensis*, *Z. montana* , and *Z. mairei*, all of which exhibited seed dimorphism (Figure 2). Based on carpel number, two morphs were distinguished: Type A (2‐carpel) and Type B (3‐carpel). The proportions of Type A seeds in *Z. xiangchengensis*, *Z. montana* , *Z. mairei*, and *Z. mauritiana* are 94.8% ± 2.71%, 77.33% ± 3.88%, 66% ± 4.54%, and 100% ± 0.00%, respectively.

**FIGURE 2:**
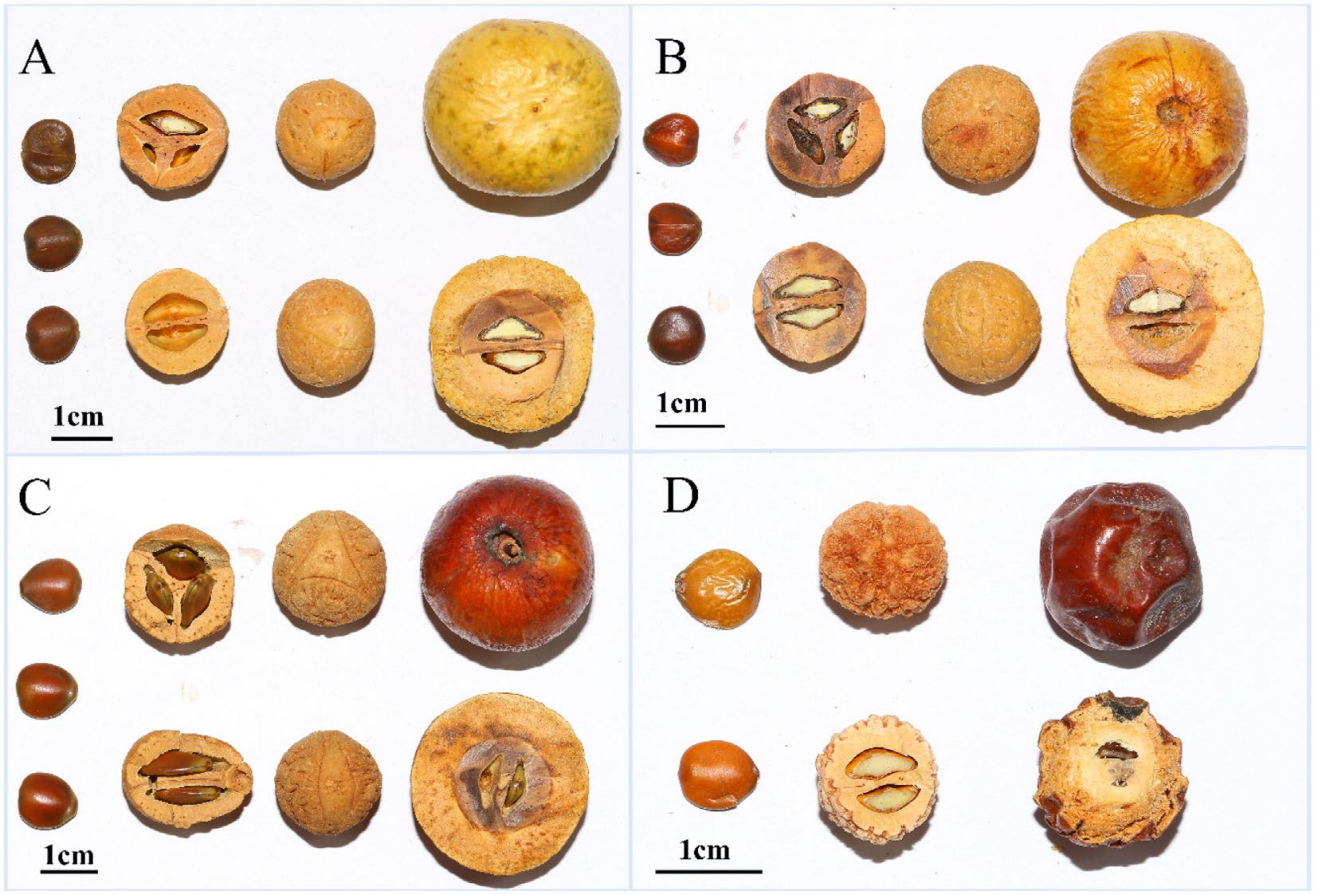
The seeds, endocarps, fruits, and cross‐sections of four *Ziziphus* species. (A) *Z. xiangchengensis*; (B) *Z. montana* ; (C) *Z. mairei*; (D) *Z. mauritiana* .

### 3.3 Effect of Temperature on the Germination of Four *Ziziphus* Species

The germination rates of the four *Ziziphus* species under the constant 20°C and alternating 15/25°C regimes are shown in Figure 3. Under constant 20°C, Type B seeds of *Z. xiangchengensis* germinated at a significantly higher rate than Type A seeds (23.33% ± 1.67% vs. 6.84% ± 1.84%; *p* < 0.05) In contrast, Type A seeds of *Z. mairei* outperformed Type B seeds (24.07% ± 3.03% vs. 10.35% ± 3.04%; *p* < 0.05). *Z. montana* showed no significant seed‐type difference, with all germination rates below 10%.

**FIGURE 3:**
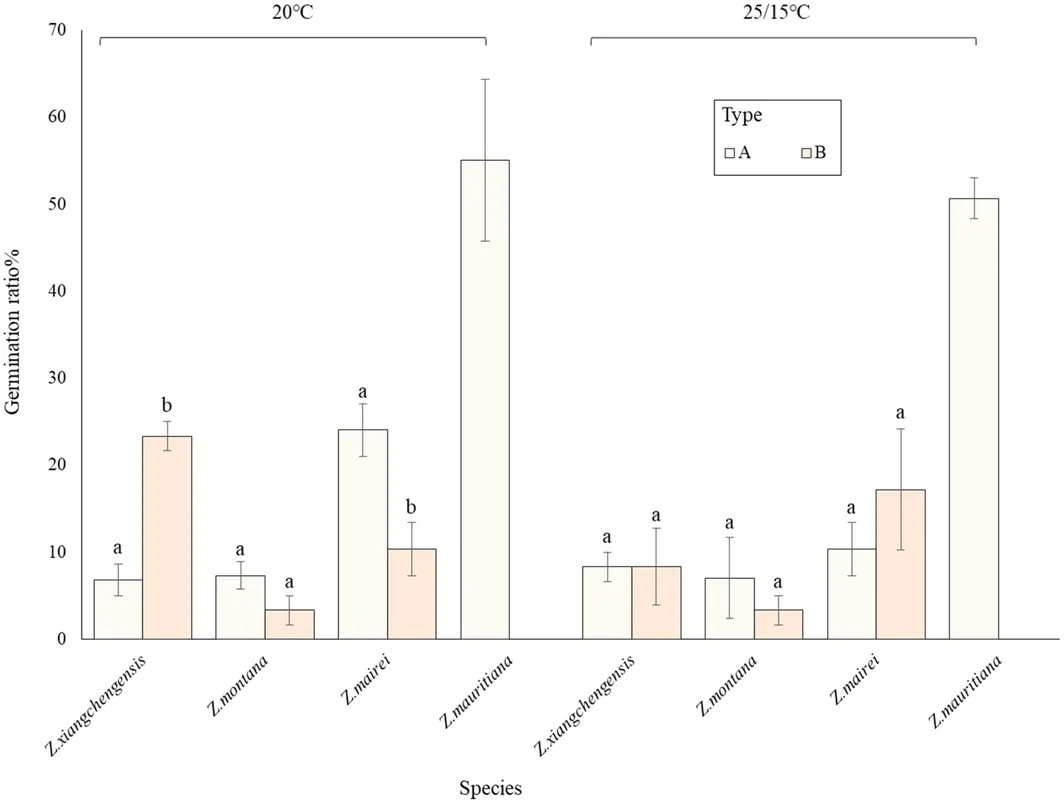
Germination rates of four *Ziziphus* species under constant (20°C) and alternating (15/25°C) temperature regimes.

Under the alternating 25/15°C regime, no significant differences in germination were observed between seed types for the three heteromorphic species, with all rates remaining below 20%. *Z. mauritiana* consistently exceeded 50% germination across both regimes. Two‐way ANOVA revealed significant interspecific differences in germination responses to seed type, temperature, and their interaction (Table S4). For *Z. xiangchengensis*, both main effects and their interaction significantly influenced germination. For *Z. mairei*, only the interaction effect was significant, while *Z. montana* showed no significant response to either factor or their interaction. The generally low germination rates observed for *Z. xiangchengensis*, *Z. montana* , and *Z. mairei* suggest the presence of seed dormancy, which was further investigated through dormancy‐breaking experiments.

### 3.4 Breaking Dormancy of Four *Ziziphus* Species

Scarification resulted in 100% germination across all four species (Table 3). Constant temperature conditions (20°C) were more conducive to germination than the alternating regime for *Z. xiangchengensis*, *Z. montana* , and *Z. mauritiana* . For *Z. xiangchengensis*, scarification significantly accelerated and synchronized germination (Table S5), whereas all others produced consistently low germination rates (< 15%). The type B seeds exhibited a slight intrinsic germination advantage over type A and initiated germination significantly earlier (Table S5).

**TABLE 3:** Germination percentages of Type A and Type B seeds of four *Ziziphus* species under various treatments at 20°C and 15/25°C.

			Blank control	1% GA_3_	70°C hot water	Scarification	Storage	Goat digestion
*Ziziphus xiangchengensis*	A	20°C	6.84 ± 1.84c	13.68 ± 3.16b	7.02 ± 1.75c	100.00 ± 0.00a	—	—
		15/25°C	8.33 ± 1.67b	3.33 ± 1.67c	—	100.00 ± 0.00a	—	—
	B	20°C	23.33 ± 1.67b	11.67 ± 4.41c	—	100.00 ± 0.00a	—	—
		15/25°C	8.33 ± 4.41b	11.67 ± 4.41b	—	100.00 ± 0.00a	—	—
*Ziziphus montana*	A	20°C	7.32 ± 1.61 cd	1.85 ± 1.85d	12.90 ± 6.52bc	100.00 ± 0.00a	7.53 ± 2.12 cd	18.34 ± 3.47b
		15/25°C	7.02 ± 4.64c	1.75 ± 1.75c	—	100.00 ± 0.00a	7.53 ± 2.12bc	16.37 ± 3.22b
	B	20°C	3.33 ± 1.67c	5.00 ± 00bc	—	100.00 ± 0.00a	11.75 ± 4.34b	—
		15/25°C	3.33 ± 1.67b	3.33 ± 3.33b	—	100.00 ± 0.00a	5.18 ± 3.04b	—
*Ziziphus mairei*	A	20°C	24.07 ± 3.03c	15.26 ± 2.90c	80.00 ± 8.67b	100.00 ± 0.00a	6.67 ± 4.41d	—
		15/25°C	10.35 ± 3.04c	22.57 ± 3.92b	—	100.00 ± 0.00a	6.67 ± 3.33a	—
	B	20°C	10.35 ± 3.04b	14.55 ± 6.72b	—	100.00 ± 0.00a	6.67 ± 3.33b	—
		15/25°C	17.22 ± 7.00b	24.56 ± 8.77b	—	100.00 ± 0.00a	10.00 ± 5.77b	—
*Ziziphus mauritiana*	A	20°C	55.06 ± 9.34c	92.45 ± 2.12ab	64.68 ± 16.18bc	100.00 ± 0.00a	—	—
		15/25°C	50.69 ± 2.32b	30.00 ± 5.25c	—	100.00 ± 0.00a	—	—

*Note:* Data are presented as mean ± SD Different lowercase letters indicate significant differences in germination percentage among treatments for the same seed type at the same temperature (*p* < 0.05). “—” indicates no experiment was conducted.

The dormancy‐breaking process in *Z. montana* was minimally modulated by environmental temperature and seed type (Table 3). Moreover, its dormancy was largely unaffected by conventional treatments involving hormones, heat shock, or after‐ripening, indicating that physiological dormancy did not play a dominant role. In *Z. montana* , only scarification and goat digestive passage produced stable and significant germination responses (100% and 16.37%–18.34%, respectively). The scarification treatment significantly accelerated the germination process, whereas germination under other treatments was slow and asynchronous (Table S6). These results indicate that the dormancy of *Z. montana* is primarily physical rather than physiological, as conventional physiological dormancy‐breaking treatments were largely ineffective. The positive but moderate effect of goat digestion suggests that endozoochory may contribute to natural regeneration, though it is insufficient for large‐scale seedling production.

For *Z. mairei*, germination responses varied markedly across treatments, seed types, and temperatures. At constant 20°C, hot water treatment (70°C) achieved the highest germination rate for Type A seeds (80.00% ± 8.67%), significantly exceeding both the control (24.07% ± 3.03%) and the 1% GA_3_ treatment (15.26% ± 2.90%) (*p* < 0.05). This indicates that heat shock effectively alleviates physiological dormancy, though the limited effect of GA_3_ suggests that dormancy is not solely regulated via the gibberellin pathway. At 20°C, the Type A seeds exhibited a significantly higher inherent germination potential (24.07%) compared to Type B (10.35%), indicating shallower dormancy and earlier germination initiation (Table S7). Alternating temperature conditions were more conducive to breaking dormancy and enhancing germination in *Z. mairei*. Its dormancy is classified as a combination of physical and physiological dormancy, the release of which strongly depends on temperature signals, including heat shock or alternating temperatures.

Among the four species, *Z. mauritiana* exhibited the shallowest dormancy, with germination exceeding 50% even in the untreated control across both temperature regimes. Under constant 20°C, the germination rate reached 92.45% with 1% GA_3_ treatment, while seed coat scarification achieved 100% germination. This confirms that *Z. mauritiana* exhibits both superficial physical dormancy and pronounced physiological dormancy. The response to GA_3_ was strictly temperature‐dependent, being effective only under constant 20°C but not under alternating temperatures. At constant temperature, germination also commenced earlier and was completed within a shorter period (Table S8). Collectively, *Ziziphus mauritiana* demonstrates a tendency toward “opportunistic germination,” capable of rapid germination under favorable conditions while maintaining physical dormancy as a safeguard against unfavorable environments.

### 3.5 Population Renewal Strategy of *Ziziphus montana*

#### 3.5.1 Age Structure of *Ziziphus montana* Populations

A total of 573 individuals were recorded within the quadrats, of which 48.21% were current‐year seedlings. The age structure of the *Z. montana* population (excluding current‐year seedlings) is presented in Figure 4. The age structure of this population is foundationally expanding. A sharp decline from current‐year seedlings to Age Class I indicates a severe mortality bottleneck during the transition from seedling establishment to sapling survival. The likely causes are browsing by livestock such as goats and the extreme climatic conditions of the Jinsha River region. Age Class II is the absolute dominant stage, and the sharp decline from Class II to Class III may result from intensified intraspecific competition and environmental filtering processes. The stable decline from Age Class III to VI suggests the presence of sustained but non‐catastrophic pressures, such as logging and natural disturbances. Age Class IV begins to mature and bear fruit. The extremely low proportion of individuals in Age Class VII and above may be associated with the legacy effect of historical logging. Most individuals in Age Class VII and above are trees attaining heights of 7–9 m, which may be an adaptation to wind resistance.

**FIGURE 4:**
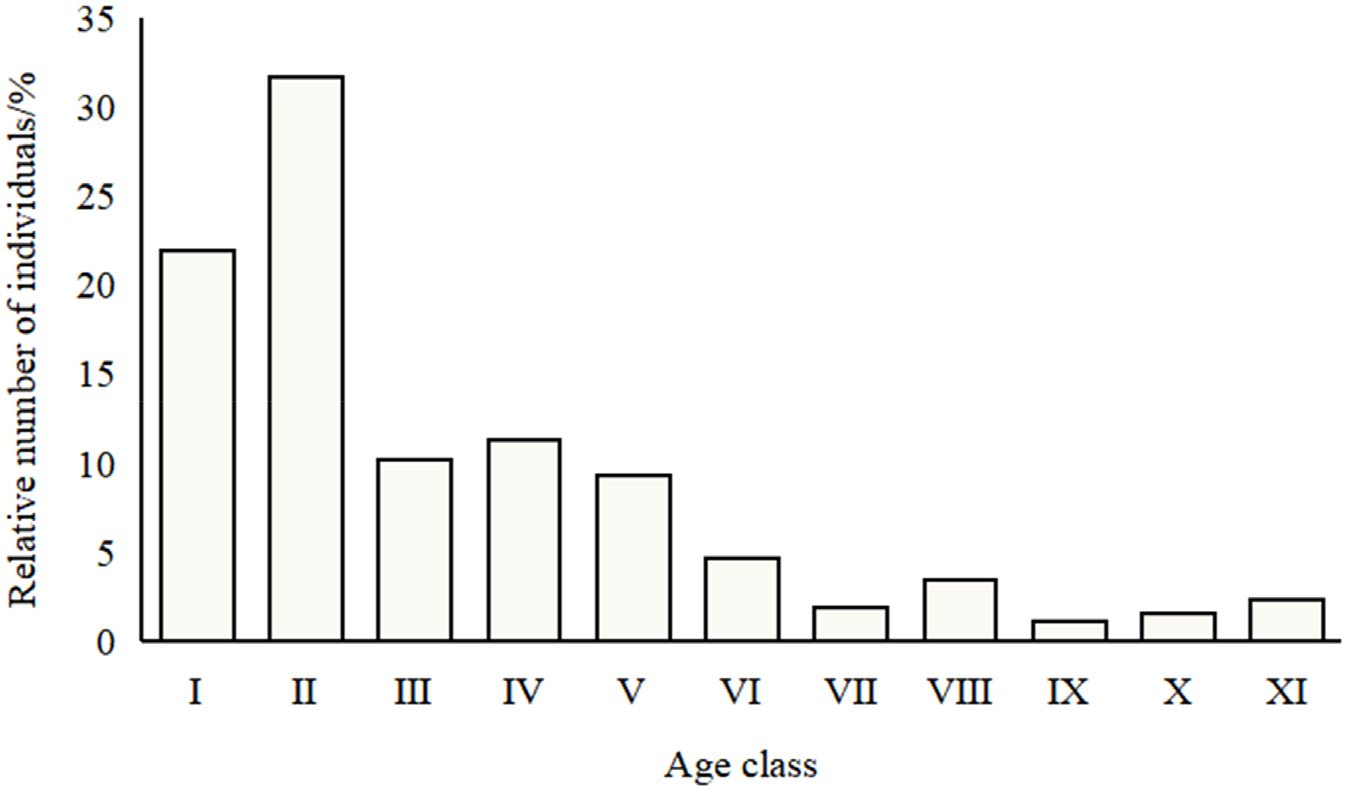
Population age structure of *Z. montana* . Age classes were defined based on ground diameter (GD) using 5 cm intervals: Class I (GD < 5 cm), Class II (5 cm ≤ GD < 10 cm), and so forth.

#### 3.5.2 Community Participation Survey Results

A total of 55 valid interviews were conducted, with 83.64% of participants aged 40 years or older. The interview results indicate that: (1) The vernacular names of *Z. montana* reflect its uses and morphology: “Bodhi seeds” is influenced by Tibetan Buddhism (Figure 5A); “vajra seeds” stems from the traditional use of its hard kernels as marbles in childhood games among the Naxi people; and “thorny disc” refers to the plant's spiny morphology. (2) *Z. montana* serves multiple purposes, including food, medicine, handicrafts, hedges, and timber. The fruit pulp possesses a tart flavor, while roasted seeds are consumed as a snack similar to sunflower seeds (Figure 5B). According to the Compendium of Chinese Ethnic Materia Medica, the seeds are used to treat infertility, and local communities also attribute hypotensive properties to them. The bony endocarp is crafted into Buddhist prayer beads or bracelets, commonly known as “Bodhi seeds.” The spiny plants are used as hedge to enclose livestock; the hard wood is employed to make farming tools. (3) Approximately two decades ago, the government initiated a ban on logging, yet has permitted activities such as animal husbandry and productive agriculture. The 70% respondents indicated that the decline in plant populations was due to logging, while 58.18% reported having observed a small number of seedlings. The high market value of “Bodhi seeds,” local residents have begun attempting to manage and cultivate wild plants. (4) Cattle and goats act as seed dispersers for *Z. montana* . Among the respondents, 98.18% have observed goats consuming both its fruits and seedlings, while 34.55% reported seeing cattle feed on the fruits.

**FIGURE 5:**
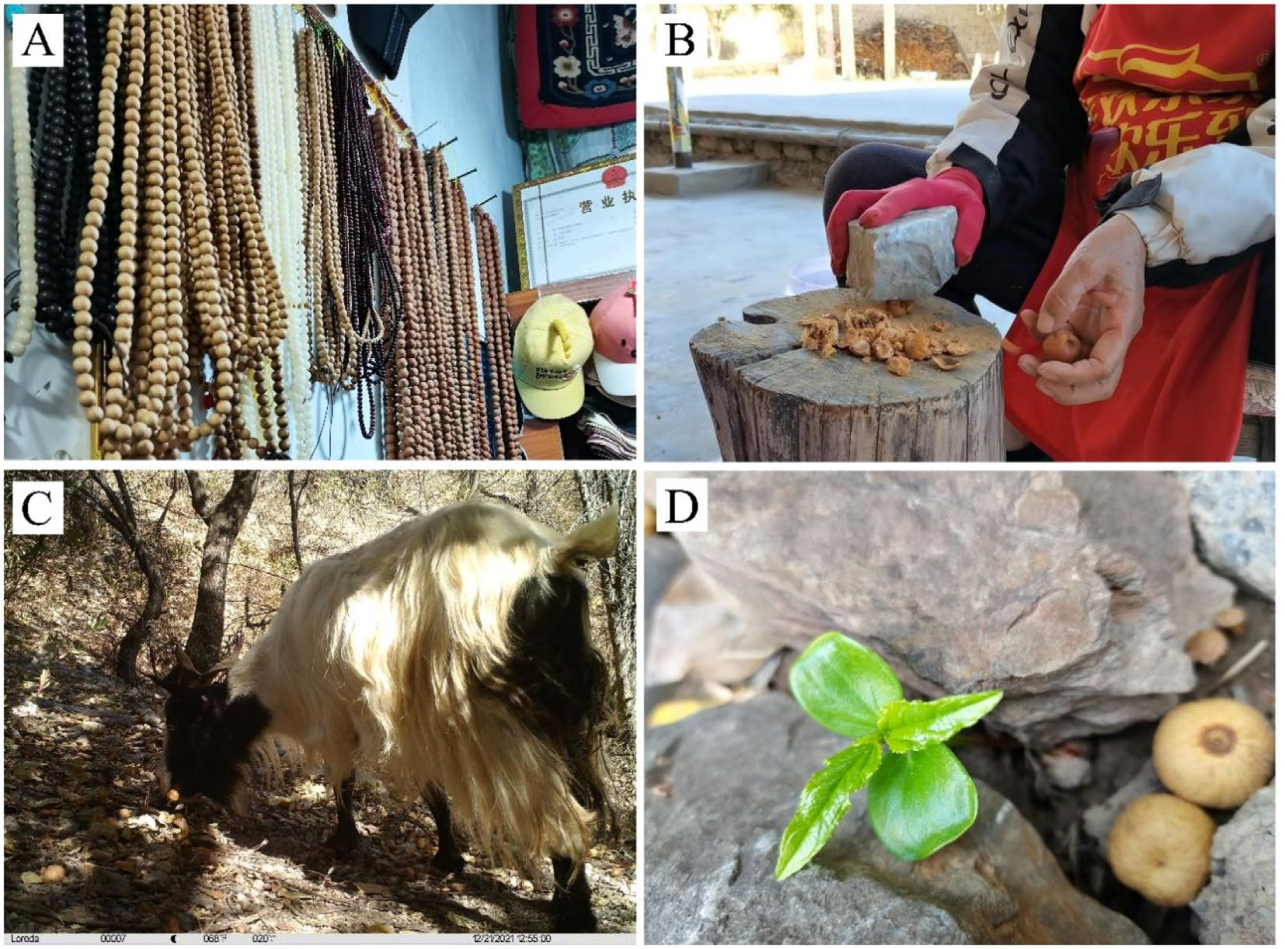
(A) the Bodhi seed bracelets crafted from the endocarps of *Z. montana* ; (B) a local resident has been collecting seeds of *Z. montana* ; (C) a goat feeding on the fruits of *Z. montana* ; (D) a seedling of *Z. montana* .

#### 3.5.3 Seed Dispersal of *Ziziphus montana*

The regeneration of *Z. montana* occurs both through seeds (Figure 5D) and sprouting, with abundant seed production serving as the primary source. Most fruits accumulate beneath the parent plant canopy, whereas those on slopes can disperse downward with the aid of gravity. Fruits located near rivers can be dispersed by water flow, owing to the impermeable exocarp and spongy mesocarp, which allow the fruits to float.

Four infrared cameras provided 304 effective monitoring days. Goats exhibited the highest frequency of feeding (Figure 5C), with 363 photographs, 212 video clips, and 204 independent detections recorded. The activity index reached 0.67, and the peak feeding period occurred between 10:00 and 13:00. The red‐bellied squirrel ( *Callosciurus erythraeus* ) was observed feeding in situ, with 7 independent detections recorded (21 photographs, 7 video clips). In addition, incidental records included human activity, as well as the presence of *Prionailurus bengalensis* and *Phoenicurus auroreus* . These findings collectively indicate that goats serve as the primary seed dispersers of *Z. montana* , exhibiting a capacity for long‐distance seed dispersal that is closely linked to human pastoral activities.

## 4 Discussion

### 4.1 Responses of Four *Ziziphus* Species to Climate Change

As a widely distributed species, *Ziziphus mauritiana* is primarily limited by mean temperature of the coldest quarter (bio11). Its northern and high‐altitudinal range limits are dictated by winter low‐temperature tolerance, consistent with low‐temperature restrictions reported for *Z. spina‐christi* (Behzadi et al. 2025). This also confirms that *Z. mauritiana* , extending from tropical Asia to Australia and Africa, is capable of adapting to a broad climatic gradient from humid to arid tropics (Yang et al. 2024). The minor contributions of bio4 and bio3 (< 7%) may reflect the strong adaptability of *Z. mauritiana* to temperature variations as a eurytopic species. Under future climate scenarios, both highly and marginally suitable habitats for *Z. mauritiana* are projected to expand substantially, potentially shifting its northern range boundary. However, extreme low‐temperature events may impact peripheral populations, and *Z. mauritiana* populations in northwestern Yunnan warrant priority for genetic diversity monitoring as germplasm resources. However, extreme cold events may still threaten peripheral populations, particularly those in northwestern Yunnan, which warrant priority monitoring as genetic resources for conservation and breeding.

For *Z. montana* , temperature seasonality (bio4) was identified as the dominant determinant of its currently highly suitable habitats, which are concentrated in the Jinsha, Lancang, and Yalong River basins. This likely reflects the strong adaptation to the extreme and regular seasonal temperature patterns that characterize these river basins. Future climate projections indicate a contraction of highly suitable areas for *Z. montana* into the three river basins, suggesting that these watersheds may serve as climatic refugia and priority zones for ecological restoration.

Predicting future plant distributions can inform climate change adaptation and guide scientific cultivation and precision management (Franklin 2010). Fragile arid ecosystems are more sensitive to climate change than other regions, with plants experiencing greater impacts. Our findings show that *Z. montana* , with its narrow habitat range, is more susceptible to environmental changes than the widely distributed *Z. mauritiana* . Previous studies have shown that the distribution of *Ziziphus* species is primarily influenced by hydrothermal conditions. For example, *Z. spina‐christi* is highly tolerant to heat and drought, with the maximum temperature of the warmest month as the primary factor determining its distribution (Behzadi et al. 2025). *Z. nummularia* is determined by the precipitation of the wettest month, and its habitats are characterized by greater diversity (Behzadi et al. 2025). *Z. jujuba* is influenced by annual mean temperature, and it is projected to expand into mid‐to‐high latitude regions under climate change (Zhao et al. 2021). While *Z. lotus* is limited by precipitation of the driest quarter (Liu et al. 2025). This study is primarily based on climatic factors for modeling and has not yet incorporated variables such as topography, soil, human activities, and biotic interactions, which are also important drivers constraining species distribution (Sun et al. 2020; Behzadi et al. 2025). Future studies integrating these variables would refine habitat suitability assessments and restoration planning.

### 4.2 Ecological Significance of Heteromorphic Seeds in *Ziziphus*: Number, Germination, and Dormancy

The seed heteromorphism was observed in *Z. mairei*, *Z. montana* , and *Z. xiangchengensis*, all of which belong to the same phylogenetic clade within the Rhamnaceae and share highly similar habitats (Li et al. 2010). In contrast, the widely distributed *Z. mauritiana* does not exhibit seed number variation and is more distantly related. The observed variation in seed number may therefore have both ecological and phylogenetic underpinnings.

The dominance of Type A seeds in all three heteromorphic species suggests a maternal resource allocation trade‐off. This numerical asymmetry may function as a “mild bet‐hedging strategy” against environmental uncertainty. When resources are abundant, increasing seed number maximizes offspring output; when resources are limited, ensuring individual seed quality enhances germination competitiveness (Harper et al. 1970). Seed number and quality are influenced by provenance and population size (Gao et al. 2010). Seed number variation in *Z. xiangchengensis* is limited (Type A: 94.8% ± 2.71%), possibly due to its small‐scale aggregated distribution pattern. Studies on other rare species have shown that small populations often exhibit reduced seed number and trait scores, yet their germination fitness is not necessarily the lowest (Gao et al. 2010). The variation in seed number may also represent an adaptation of these three endemic plants to the highly heterogeneous environments of dry river valleys.

Beyond numerical variation, seed traits may also reflect adaptations for dispersal, dormancy, or other functions (Gao et al. 2010). Temperature is a key regulator of germination (Bewley and Black 1994), and our experiments revealed contrasting germination strategies among the three heteromorphic species. In *Z. xiangchengensis*, the dominant Type A seeds (94.8%) adopt a stable “conservative strategy,” maintaining low germination rates (< 10%) under both temperature regimes, while the minor Type B seeds (5.2%) represent an “opportunistic strategy,” with earlier germination initiation but greater sensitivity to alternating temperatures (inhibit germination). In contrast, *Z. mairei* exhibits the opposite strategy: Type A seeds (66%) are the opportunistic dominant with earlier germination, while Type B seeds (34%) function as the ample reserve. For *Z. montana* , both seed types adopt a “conservative strategy,” with low germination rates under both temperature regimes, though Type A seeds exhibited higher baseline germination.

The physical dormancy of the four *Ziziphus* species is caused by the impermeability of the stony endocarp and seed coat, consistent with other *Ziziphus* species (Dorji et al. 2015). However, physiological dormancy varied among species and seed types. In *Z. xiangchengensis*, Type A seeds exhibited GA‐deficient physiological dormancy, whereas Type B seeds had sufficient endogenous GA_3_. Under alternating 15/25°C conditions, no promoting effect of GA_3_ was observed, indicating that temperature regulation of germination is not primarily mediated through the GA pathway. In *Z. montana* , Type A seeds were strongly inhibited by GA_3_ treatment (suggesting sufficient endogenous GA_3_), whereas Type B seeds showed slight promotion. In *Z. mairei*, Type A seeds possess sufficient endogenous GA_3_, yet their germination is inhibited by alternating temperatures. However, this inhibition can be alleviated by exogenous GA_3_ application. Type B seeds were insensitive to both temperature and GA_3_ treatments.

### 4.3 Methods for Breaking Dormancy in Four *Ziziphus* Species

Physical dormancy, imposed by the impermeable stony endocarp and seed coat, is the primary constraint on germination across all four species. Scarification achieved 100% germination in all species, confirming that physical dormancy can be readily overcome by simple treatment for large‐scale seedling production.

Physiological dormancy also varies among different species and seed types of *Ziziphus* species. Exogenous GA_3_ significantly promoted germination only in *Z. mauritiana* , suggesting a GA‐deficient component in this species. In contrast, Type B seeds of *Z. xiangchengensis*, both seed types of *Z. montana* , Type A seeds of *Z. mairei*, and *Z. mauritiana* all contain sufficient endogenous GA_3_, as exogenous application produced no additional benefit. Germination requirements vary considerably among *Ziziphus* species. *Ziziphus mucronata* lacks embryo dormancy and tolerates prolonged water stress (Zietsman and Botha 1987), whereas *Z. lotus* requires 120 days of cold stratification to achieve 83% germination (Kheloufi et al. 2020). *Ziziphus mauritiana* has a high optimal germination temperature, with 77% germination recorded at 33/27°C (Bebawi et al. 2016), whereas low temperatures induce temporary dormancy (Kricsfalusy and Mező‐Kricsfalusy 1994). Optimal germination temperatures may be relatively high across *Ziziphus* species, with 35°C reported as optimal for *Z. lotus* (Maraghni et al. 2010). The contrasting germination strategies among these species highlight the need for endogenous hormone profiling to further validate the physiological mechanisms underlying these differences.

Germination responses to seed storage varied by species and seed type. After 1 year of storage, the germination rate of Type A seeds of *Z. montana* did not increase, while Type B seeds showed a slight improvement. Germination of both seed types in *Z. mairei* declined, indicating potentially short seed longevity. Such variation is consistent with reports for other *Ziziphus* species: *Z. budhensis* seeds remain viable for only 4 months under dry and cool storage conditions (Dorji et al. 2015), whereas *Z. mauritiana* seeds remain viable for 18–24 months, suggesting a short‐term persistent seed bank. In *Z. escuba*, by contrast, germination increased after 4 months of storage (Paudel et al. 2023). Future research will examine how storage duration affects seed viability in the four *Ziziphus* species, aiming to evaluate their potential for long‐term germination in the field.

Heat shock significantly promoted germination only in *Z. montana* seeds, with no detectable effects on the other species. *Ziziphus mucronata* also showed no significant response to boiling water treatment (Hassen et al. 2005). Besides, passage through the goat digestive tract significantly enhanced germination in *Z. montana* , indicating effective endozoochorous dispersal. This suggests that dormancy in *Z. montana* is an adaptation for endozoochorous dispersal, and that goats, which are ubiquitous in the Jinsha River basin, act as both consumers and dispersers of this species. In addition to the factors discussed above, sowing depth (burial), water availability, seed maturity, and storage conditions also significantly influence germination in *Ziziphus* species (Maraghni et al. 2010; Bebawi et al. 2016). Notably, under natural conditions, fruits of *Ziziphus* species located on the soil surface exhibit higher germination rates than those buried beneath the soil. The hard endocarp may be abraded by wind, water, temperature changes, microbes, and animal gut passage, while buried seeds face greater risk from pathogens and pests (Dorji et al. 2015). These factors should be considered in restoration practice to optimize seedling establishment.

### 4.4 Ecological Restoration Potential of *Ziziphus montana* in the Jinsha River Basin and Its Significance

Based on our assessment, *Z. montana* is highly suitable for ecological restoration in the Jinsha River Basin. This recommendation is supported by the following reasons: (1) it has more extant populations than the rarer *Z. xiangchengensis* and *Z. mairei*, and its highly suitable habitats are concentrated within the Jinsha River basin; (2) under future climate scenarios, the Jinsha River Basin will remain a highly suitable area for *Z. montana* , making it less vulnerable to environmental changes; (3) mature individuals produce abundant fruits, providing capacity for seedling recruitment; (4) scarification achieves 100% germination, enabling large‐scale seedling production; (5) livestock (cattle and goats) act as effective seed dispersers in the grazing ecosystems of the region; and (6) *Z. montana* has high economic and cultural value, with diverse uses including food, medicine, handicrafts, hedgerows, and timber.

The harsh climatic, topographical, and anthropogenic conditions of the Jinsha River Basin make ecological restoration particularly challenging. We advocate for community‐based “interactive” ecological restoration, using economic incentives to drive ecological recovery, thereby achieving a win‐win outcome. While livestock grazing promotes *Z. montana* seed dispersal, careful management is needed to prevent seedling damage from cattle and goats. The potential economic value of *Z. montana* requires validation through accurate chemical analysis of its fruits, leaves, and seeds. Finally, long‐term monitoring of *Z. montana* growth is needed to evaluate the effectiveness of using this species for restoration.

### 4.5 Methodological Transferability and Broader Implications

This study integrates a comprehensive framework of species distribution modeling, germination experiments, and field investigations, which can be widely applied to other ecological restoration scenarios in arid areas where native species are preferred for ecological restoration. Although the empirical data are specific to *Ziziphus* species in the Jinsha River basin, the four‐step approach can be adapted for other regions and taxa: (1) species‐site matching under climate change using MaxEnt, and select highly suitable species and restoration sites; (2) seed germination characterization to understand dormancy types and temperature responses, and to develop practical protocols for rapid, large‐scale seedling production; (3) field assessment of natural regeneration and value of the target species via population structure analysis, seed disperser monitoring, and community interviews; and (4) community‐engaged restoration driven by economic incentives tailored to local socioeconomic conditions. While species‐specific validation is required before applying dormancy‐breaking protocols to other taxa, and incentive mechanisms must be adapted to local contexts, the multi‐evidence framework provides a reproducible template for evaluating restoration potential of native species in data‐scarce regions.

## 5 Conclusions

This study assessed the restoration potential of four *Ziziphus* species in the Jinsha River basin through species distribution modeling, germination experiments, and field surveys. This study reports, for the first time, seed dimorphism in *Z. xiangchengensis*, *Z. montana* , and *Z. mairei*, with Type A seeds being dominant (66.0%–94.8%). The two seed types differed significantly in both seed number (intraspecific variation) and physiological traits, including germination and dormancy, and this variation may be attributed to adaptation to highly heterogeneous environments or phylogeny. Scarification achieved 100% germination, confirming that the physical dormancy bottleneck can be readily overcome for large‐scale seedling production. Under future climate scenarios, suitable habitat for *Z. mauritiana* is projected to expand (44.1%–119.1%), whereas that of *Z. montana* is projected to contract (11.3%–20.4%). However, the Jinsha River basin remains a highly suitable habitat for *Z. montana* and has strong potential for recovery. Field surveys revealed larger *Z. montana* populations than *Z. mairei* and *Z. xiangchengensis*, with an expanding age structure but a critical seedling‐to‐sapling bottleneck, likely due to grazing and aridity. Local interviews documented diverse uses and economic value, providing incentives for community‐based restoration. This study recommends designating *Z. montana* as a priority species for ecological restoration in the Jinsha River basin and implementing targeted measures, including fencing off seedling areas, protecting seed trees, and promoting community participation through economic incentives. Future research should incorporate non‐climatic variables, conduct long‐term monitoring, and verify the economic potential of *Z. montana* products. This study provides valuable insights for ecological restoration in the Jinsha River Basin, and holds positive implications for the conservation of the four wild *Ziziphus* species' germplasm resources and for local economic development.

## Author Contributions

**Yulin Xu:** data curation (equal), methodology (equal), resources (equal), software (equal), writing – original draft (equal), writing – review and editing (equal). **Zaiqiu Xiong:** data curation (equal), investigation (equal), methodology (equal), writing – original draft (equal), writing – review and editing (equal). **Zhiming Zhang:** data curation (equal), resources (equal). **Gao Chen:** conceptualization (lead), funding acquisition (lead), project administration (lead), validation (equal), visualization (equal).

## Funding

This work was equally supported by the National Key R&D Program of China (2024YFF1306700), the Regional Innovative Development Joint Fund of NSFC (U23A20149), the Key Project of Basic Research of Yunnan Province, China (202301AS070001), and the Key Industrial Science and Technology Projects Served by Yunnan Province's Universities (FWCY‐BSPY2025014).

## Conflicts of Interest

The authors declare no conflicts of interest.

## Supporting information


**Figure S1:** The results of the jackknife test of variable importance. (A–C) *Ziziphus mauritiana* ; (D–F) *Z. montana*.
**Table S1:** The fruit collection and habitat information for four *Ziziphus* species.
**Table S2:** Content of the Questionnaire.
**Table S3:** The AUC values of the training and testing sets of the MaxEnt models.
**Table S4:** Two‐way ANOVA results of the effects of seed type and temperature on germination rate for three *Ziziphus* species.
**Table S5:** Germination Characteristics of Type A and Type B Seeds of *Z. xiangchengensis* under Conditions of 20°C and 25/15°C.
**Table S6:** Germination Characteristics of Type A and Type B Seeds of *Z. montana* under Conditions of 20°C and 25/15°C.
**Table S7:** Germination Characteristics of Type A and Type B Seeds of *Z. mairei* under Conditions of 20°C and 25/15°C.
**Table S8:** Germination Characteristics of Type A Seeds of *Z. mauritiana* under Conditions of 20°C and 25/15°C.

## Data Availability

All the required data are uploaded as Supporting Information S1.
