# Supplementary material for: Evaluating the Potential of Four Native Ziziphus Species for Restoring Savanna‐Like Habitats in Jinsha River Under Climate Change
Source: Ecol Evol. 2026 Oct 28;16(11):e74531. doi: 10.1002/ece3.74531 (PMC13714631; doi:10.1002/ece3.74531)
Supplement: Supplementary file 1 [file ECE3-16-e74531-s001.docx]

**Evaluating the potential of four native *Ziziphus* species for restoring savanna-like habitats in Jinsha River under climate change**

**Figure captions**

**Figure S1** The results of the jackknife test of variable importance.

**Figure S1** The results of the jackknife test of variable importance. (A-C) *Ziziphus mauritiana*; (D-F) *Z. montana.*

**Table captions**

**Table S1** The fruit collection and habitat information for four *Ziziphus* species.

**Table S2** Content of the Questionnaire.

**Table S3** The AUC values of the training and testing sets of the MaxEnt models.

**Table S4** Two-way ANOVA results of the effects of seed type and temperature on germination rate for three *Ziziphus* species.

**Table S5** Germination Characteristics of Type A and Type B Seeds of *Z. xiangchengensis* under Conditions of 20°C and 25/15°C

**Table S6** Germination Characteristics of Type A and Type B Seeds of *Z. montana* under Conditions of 20°C and 25/15°C

**Table S7** Germination Characteristics of Type A and Type B Seeds of *Z. mairei* under Conditions of 20°C and 25/15°C

**Table S8** Germination Characteristics of Type A Seeds of *Z. mauritiana* under Conditions of 20°C and 25/15°C

**Table S1** The fruit collection and habitat information for four *Ziziphus* species.

| Species | Latitude (N) | Longitude (E) | Altitude (m) | Collection Time | Habitat |
| --- | --- | --- | --- | --- | --- |
| *Z. mauritiana* | 25.8551° | 101.8484° | 1071 | 2021 | dry-hot valley open forest |
| *Z. montana* | 27.2880° | 100.2385° | 1769 | 2020 | in the rocky site of the dry-hot valley open forest |
|  | 27.4908° | 100.0763° | 2333 | 2021 |  |
| *Z. xiangchengensis* | 28.8909° | 99.8233° | 2773 | 2021 | in the rocky site of the dry-hot valley open forest |
| *Z. mairei* | 25.1347° | 102.7422° | 1959 | 2021, 2022 | forest habitat |

**Table S2** Content of the Questionnaire.

| Question | Details |
| --- | --- |
| Question 1 | Have you seen *Z. montana*? |
| Question 2 | What is the local name for *Z. montana*? |
| Question 3 | How is this plant used here? Are any cultivation practices presently employed for this species? |
| Question 4 | Will agricultural or livestock activities take place near the plants? Will cattle, sheep, or other animals consume the fruits or leaves? |
| Question 5 | Compared to before, do you think the number of plants has increased or decreased? What do you think might be the reason? |
| Question 6 | Are there any governmental regulations or restrictions in place that prohibit the cutting of this species? |

**Table S3** The AUC values of the training and testing sets of the MaxEnt models

|  |  | Training AUC | Testing AUC |
| --- | --- | --- | --- |
| *Z. mauritiana* | Current | 0.977 | 0.969 |
|  | SSP126-2050S | 0.977 | 0.969 |
|  | SSP585-2050S | 0.976 | 0.969 |
| *Z. montana* | Current | 0.987 | 0.981 |
|  | SSP126-2050S | 0.986 | 0.982 |
|  | SSP585-2050S | 0.987 | 0.979 |

**Table S4** Two-way ANOVA results of the effects of seed type and temperature on germination rate for three *Ziziphus* species.

|  | Source of Variation | Degrees of freedom | Mean Square | F-value | P-value |
| --- | --- | --- | --- | --- | --- |
| *Z. xiangchengensis* | Seed Type | 1 | 204 | 9.578 | 0.0148* |
|  | Temperature | 1 | 136.9 | 6.427 | 0.035* |
|  | Seed Type × Temperature | 1 | 204 | 9.578 | 0.0148* |
| *Z. montana* | Seed Type | 1 | 44.15 | 1.983 | 0.197 |
|  | Temperature | 1 | 0.07 | 0.003 | 0.957 |
|  | Seed Type × Temperature | 1 | 0.07 | 0.003 | 0.957 |
| *Z. mairei* | Seed Type | 1 | 35.2 | 0.612 | 0.4567 |
|  | Temperature | 1 | 35.2 | 0.612 | 0.4567 |
|  | Seed Type × Temperature | 1 | 318.2 | 5.53 | 0.0466 * |

Note: * indicates a significant effect at the 0.05 level.

**Table S5** Germination Characteristics of Type A and Type B Seeds of *Z. xiangchengensis* under Conditions of 20°C and 25/15°C

| Type | temperature | Treatments | Germination characteristics | | | | |
| --- | --- | --- | --- | --- | --- | --- | --- |
|  |  |  | GP | TFG | TLG | T_50_ | CGR |
| A | 20°C | Blank control | 6.84±1.84c | 13.33±5.33a | 13.33±5.33a | 12.75±5.65a | 9.72±2.78b |
|  |  | 1% GA3 | 13.68±3.16b | 9.33±1.76a | 20.00±2.31a | 14.17±2.49a | 8.38±1.55b |
|  |  | 70°C hot water | 7.02±1.75c | 6.67±1.33a | 8.00±0.00a | 7.33±0.67a | 14.58±2.08ab |
|  |  | Scarification | 100.00±0.00a | 4.00±0.00a | 7.67±1.33a | 4.48±0.08a | 20.02±0.75a |
| B | 20°C | Blank control | 23.33±1.67b | 5.67±0.67a | 18.33±1.45a | 10.00±0.58a | 10.75±0.58b |
|  |  | 1% GA3 | 11.67±4.41c | 5.67±0.88a | 13.00±4.16ab | 8.08±0.85a | 13.74±2.08b |
|  |  | Scarification | 100.00±0.00a | 4.00±0.00a | 7.00±0.58b | 4.48±0.02b | 20.41±0.19a |
| A | 15/25°C | Blank control | 8.33±1.67b | 13.33±5.33a | 18.67±5.33a | 13.17±5.17a | 8.33±2.41a |
|  |  | 1% GA3 | 3.33±1.67c | 5.00±2.52a | 5.00±2.52b | 4.47±2.35a | 8.93±4.49a |
|  |  | Scarification | 100.00±0.00a | 5.00±0.00a | 8.33±0.88ab | 5.50±0.13a | 16.55±0.25a |
| B | 15/25°C | Blank control | 8.33±4.41b | 5.00±2.65a | 13.00±7.23a | 4.83±2.62a | 7.19±3.72b |
|  |  | 1% GA3 | 11.67±4.41b | 6.33±0.88a | 15.67±4.98a | 6.83±0.44a | 11.90±0.79ab |
|  |  | Scarification | 100.00±0.00a | 3.67±1.33a | 9.33±0.67a | 6.83±0.20a | 16.33±1.91a |

Note: Data are presented as mean ± SD. GP: Germination percentage; TFG: Time for the first germination; TLG: Time for the last germination; T_50_: Time to reach 50% of final germination. CGR: Corrected germination rate index. Different lowercase letters indicate significant differences in germination percentage with the same type seeds at the 5% level. The same as below.

**Table S6** Germination Characteristics of Type A and Type B Seeds of *Z. montana* under Conditions of 20°C and 25/15°C

| Type | temperature | Treatments | Germination characteristics | | | | |
| --- | --- | --- | --- | --- | --- | --- | --- |
|  |  |  | GP | TFG | TLG | T_50_ | CGR |
| A | 20°C | Blank control | 7.32±1.61cd | 9.67±3.18a | 10.67±2.91a | 9.33±3.11a | 11.69±3.02abc |
|  |  | 1% GA3 | 1.85±1.85d | 2.67±2.67a | 2.67±2.67a | 2.50±2.50a | 4.17±4.17c |
|  |  | Goat digestion | 18.34±3.47b | 6.33±0.88a | 11.67±3.48a | 7.67±1.23a | 13.27±1.70ab |
|  |  | 70°C hot water | 12.90±6.52bc | 7.00±4.73a | 9.67±5.04a | 7.58±4.90a | 6.63±3.98bc |
|  |  | Scarification | 100.00±0.00a | 4.00±0.00a | 7.00±0.58a | 5.68±0.04a | 18.34±0.69a |
|  |  | Storage | 7.32±1.61cd | 9.67±3.18a | 10.67±2.91a | 9.33±3.11a | 11.69±3.02abc |
| B | 20°C | Blank control | 3.33±1.67c | 8.33±4.91a | 8..33±4.91a | 8.00±4.77a | 6.13±3.61b |
|  |  | 1% GA3 | 5.00±00bc | 11.33±3.84a | 11.33±3.84a | 10.83±3.84a | 10.68±2.76ab |
|  |  | Scarification | 100.00±0.00a | 4.00±0.00a | 6.67±0.33a | 5.20±0.47a | 20.11±0.56a |
|  |  | Storage | 11.75±4.34b | 11.33±5.84a | 16.00±5.13a | 11.83±5.40a | 10.92±4.06ab |
| A | 15/25°C | Blank control | 7.02±4.64c | 11.33±5.78a | 13.67±6.89ab | 12.33±6.17a | 3.58±1.79c |
|  |  | 1% GA3 | 1.75±1.75c | 3.33±3.33a | 3.33±3.33b | 3.17±3.17a | 3.33±3.33c |
|  |  | Goat digestion | 16.37±3.22b | 8.67±0.88a | 21.00±3.00a | 13.58±4.94a | 8.35±1.16bc |
|  |  | Scarification | 100.00±0.00a | 6.00±0.00a | 8.33±0.88b | 6.46±0.05a | 14.45±0.18a |
|  |  | Storage | 7.53±2.12bc | 6.33±0.33a | 9.33±2.85ab | 6.33±0.60a | 14.21±1.44ab |
| B | 15/25°C | Blank control | 3.33±1.67b | 9.67±5.24a | 9.67±5.24a | 9.33±5.09a | 4.88±2.65ab |
|  |  | 1% GA3 | 3.33±3.33b | 8.00±8.00a | 8.33±8.33a | 8.17±8.17a | 1.36±1.36b |
|  |  | Scarification | 100.00±0.00a | 6.67±0.33a | 10.00±0.00a | 7.71±0.43a | 12.29±0.46a |
|  |  | Storage | 5.18±3.04b | 6.00±3.06a | 6.33±3.18a | 5.83±2.95a | 7.27±3.67ab |

Note: Data are presented as mean ± SD. GP: Germination percentage; TFG: Time for the first germination; TLG: Time for the last germination; T_50_: Time to reach 50% of final germination. CGR: Corrected germination rate index. Different lowercase letters indicate significant differences in germination percentage with the same type seeds at the 5% level. The same as below.

**Table S7** Germination Characteristics of Type A and Type B Seeds of *Z. mairei* under Conditions of 20°C and 25/15°C

| Type | temperature | Treatments | Germination characteristics | | | | |
| --- | --- | --- | --- | --- | --- | --- | --- |
|  |  |  | GP | TFG | TLG | T_50_ | CGR |
| A | 20°C | Blank control | 24.07±3.03c | 4.67±0.33a | 14.67±1.33ab | 5.33±0.17a | 15.62±0.61ab |
|  |  | 1% GA3 | 15.26±2.90c | 5.00±0.00a | 16.67±5.04a | 7.50±1.26a | 13.22±2.03b |
|  |  | 70°C hot water | 80.00±8.67b | 6.00±0.00a | 20.33±2.91a | 8.54±0.72a | 12.03±0.67bc |
|  |  | Scarification | 100.00±0.00a | 4.00±1.00a | 6.67±0.67b | 5.26±0.38a | 19.89±0.90a |
|  |  | Storage | 6.67±4.41d | 5.67±2.85a | 10.67±7.06ab | 10.25±6.85a | 6.33±3.61c |
| B | 20°C | Blank control | 10.35±3.04b | 9.67±4.18a | 14.00±4.58ab | 11.00±3.91a | 11.94±4.31ab |
|  |  | 1% GA3 | 14.55±6.72b | 9.33±4.84a | 18.33±1.20a | 9.92±4.39a | 11.41±3.14ab |
|  |  | Scarification | 100.00±0.00a | 4.00±0.57a | 7.00±0.00b | 4.59±0.02a | 19.62±0.24a |
|  |  | Storage | 6.67±3.33b | 6.00±3.00a | 8.67±4.67ab | 6.00±3.00a | 6.41±3.25b |
| A | 15/25°C | Blank control | 10.35±3.04c | 9.67±4.18a | 14.00±4.58a | 11.00±3.91a | 11.94±4.31a |
|  |  | 1% GA3 | 22.57±3.92b | 5.67±0.88a | 10.00±1.53a | 7.08±0.30a | 14.32±1.22a |
|  |  | Scarification | 100.00±0.00a | 6.00±0.00a | 8.33±0.88a | 6.49±0.07a | 14.45±0.18a |
|  |  | Storage | 6.67±3.33a | 6.00±3.00a | 8.67±4.67a | 6.00±3.00a | 6.41±3.25a |
| B | 15/25°C | Blank control | 17.22±7.00b | 7.33±1.45a | 10.33±1.45a | 8.58±0.68a | 11.93±1.55a |
|  |  | 1% GA3 | 24.56±8.77b | 6.00±0.58a | 17.00±3.61a | 10.00±1.76a | 10.86±1.07a |
|  |  | Scarification | 100.00±0.00a | 6.67±0.33a | 10.00±0.00a | 7.71±0.43a | 12.29±0.46a |
|  |  | Storage | 10.00±5.77b | 7.67±3.84a | 11.67±6.17a | 8.67±4.37a | 4.75±2.40b |

Note: Data are presented as mean ± SD. GP: Germination percentage; TFG: Time for the first germination; TLG: Time for the last germination; T_50_: Time to reach 50% of final germination. CGR: Corrected germination rate index. Different lowercase letters indicate significant differences in germination percentage with the same type seeds at the 5% level. The same as below.

**Table S8** Germination Characteristics of Type A Seeds of *Z. mauritiana* under Conditions of 20°C and 25/15°C

| Type | temperature | Treatments | Germination characteristics | | | | |
| --- | --- | --- | --- | --- | --- | --- | --- |
|  |  |  | GP | TFG | TLG | T_50_ | CGR |
| A | 20°C | Blank control | 55.06±9.34c | 6.00±0.58a | 22.00±2.08a | 16.53±0.83a | 7.60±0.76b |
|  |  | 1% GA3 | 92.45±2.12ab | 4.67±0.88a | 23.00±1.53a | 10.50±1.26b | 11.02±0.71b |
|  |  | 70°C hot water | 64.68±16.18bc | 6.00±0.58a | 18.67±3.48a | 12.83±1.01b | 8.93±0.56b |
|  |  | Scarification | 100.00±0.00a | 1.67±0.67b | 5.00±2.00b | 2.50±0.29c | 41.80±4.25a |
| A | 15/25°C | Blank control | 50.69±2.32b | 8.33±1.33a | 25.00±0.00a | 16.19±1.78a | 7.04±0.42b |
|  |  | 1% GA3 | 30.00±5.25c | 6.67±1.45a | 23.00±2.00a | 16.67±2.40a | 8.75±2.21b |
|  |  | Scarification | 100.00±0.00a | 1.00±0.00b | 8.33±1.20b | 4.25±0.38b | 37.47±2.07a |

Note: Data are presented as mean ± SD. GP: Germination percentage; TFG: Time for the first germination; TLG: Time for the last germination; T_50_: Time to reach 50% of final germination. CGR: Corrected germination rate index. Different lowercase letters indicate significant differences in germination percentage with the same type seeds at the 5% level. The same as below.
